# Characterization of Duck (*Anas platyrhynchos*) Short Tandem Repeat Variation by Population-Scale Genome Resequencing

**DOI:** 10.3389/fgene.2018.00520

**Published:** 2018-10-30

**Authors:** Wenlei Fan, Lingyang Xu, Hong Cheng, Ming Li, Hehe Liu, Yong Jiang, Yuming Guo, Zhengkui Zhou, Shuisheng Hou

**Affiliations:** ^1^Key Laboratory of Animal (Poultry) Genetics Breeding and Reproduction, Ministry of Agriculture and Rural Affairs, State Key Laboratory of Animal Nutrition, Institute of Animal Science, Chinese Academy of Agricultural Sciences, Beijing, China; ^2^State Key Laboratory of Animal Nutrition, College of Animal Science and Technology, China Agricultural University, Beijing, China; ^3^College of Animal Science and Technology, Northwest A&F University, Yangling, China

**Keywords:** short tandem repeat, duck, whole genome resequencing, population genetics, variation

## Abstract

Short tandem repeats (STRs) are usually associated with genetic diseases and gene regulatory functions, and are also important genetic markers for analysis of evolutionary, genetic diversity and forensic. However, for the majority of STRs in the duck genome, their population genetic properties and functional impacts remain poorly defined. Recent advent of next generation sequencing (NGS) has offered an opportunity for profiling large numbers of polymorphic STRs. Here, we reported a population-scale analysis of STR variation using genome resequencing in mallard and Pekin duck. Our analysis provided the first genome-wide duck STR reference including 198,022 STR loci with motif size of 2–6 base pairs. We observed a relatively uneven distribution of STRs in different genomic regions, which indicates that the occurrence of STRs in duck genome is not random, but undergoes a directional selection pressure. Using genome resequencing data of 23 mallard and 26 Pekin ducks, we successfully identified 89,891 polymorphic STR loci. Intensive analysis of this dataset suggested that shorter repeat motif, longer reference tract length, higher purity, and residing outside of a coding region are all associated with an increase in STR variability. STR genotypes were utilized for population genetic analysis, and the results showed that population structure and divergence patterns among population groups can be efficiently captured. In addition, comparison between Pekin duck and mallard identified 3,122 STRs with extremely divergent allele frequency, which overlapped with a set of genes related to nervous system, energy metabolism and behavior. The evolutionary analysis revealed that the genes containing divergent STRs may play important roles in phenotypic changes during duck domestication. The variation analysis of STRs in population scale provides valuable resource for future study of genetic diversity and genome evolution in duck.

## Introduction

Short tandem repeats (STRs), also known as short sequence repeats(SSRs) or microsatellites, are tandem repeat nucleotides of 1–6 bp in DNA sequences. These sequences are ubiquitously present in eukaryotic and prokaryotic genomes, and occur in both genic and intergenic regions (Toth et al., 2000). They are often highly variable with mutation rates dependent on several factors, including the STR motif length, repeat number, purity and their locations in the genome (Brandstrom and Ellegren, 2008; Payseur et al., 2011). The length polymorphism makes STRs into more informative genetic markers, hence the STRs are used extensively in varied aspects such as genetic diversity analysis (Santos et al., 2016; Ginja et al., 2017), genetic linkage mapping (Rohrer et al., 1996) and parentage analysis (Cherel et al., 2011). Emerging evidence suggests that STRs may play a regulatory role in complex traits of numerous species, including mice, dogs, and humans (Rd and Garner, 2004; Gemayel et al., 2010). STR variations in coding regions directly produce mutant proteins and cause human genetics disorders, such as Huntington disease and Fragile X syndrome (Pearson et al., 2005). The presence of STRs in promoters, 3′-untranslated regions (3′-UTR), 5′-untranslated regions (5′-UTR) and introns might regulate gene expression and function through various mechanisms (Sonay et al., 2015; Bagshaw, 2017). For instance, the copy number variation of the “CCG” trinucleotide repeat identified in the promoter of *Pleomorphic adenoma gene 1* (*PLAG1*), was proved to be the potential causative mutation influencing bovine stature by serving as nuclear factor binding sites and modulating the expression of *PLAG1* (Karim et al., 2011).

Population genetics has played an important role in exploring genetic variations in human and domestic animals (Larson and Burger, 2013). Investigating the population genetics and natural selection of STRs could enable us to understand their mutational mechanisms and functional impacts. Population scale analysis of STR variations in humans and flies has provides a genomic view of factors affecting STR polymorphism (Payseur et al., 2011; Fondon et al., 2012; Willems et al., 2014). On the other hand, population-specific STRs are usually located in candidate regions under selection and are potentially responsible for diverse phenotypes. For example, comparative genomic study of STRs in developmental genes of 92 breeds of dogs revealed that profound evolution in the snout morphology of domestic dog breeds are caused by length mutations of a compound STR in the gene *Runx2* (Rd and Garner, 2004). However, in domestic animals, most studies have limited themselves to only a few genomes or very small panels of STRs. The investigation of diversity and origin of STRs, the characterization of their population-genetic properties, and the determination of their functional impacts are still active areas of research.

The advances of next generation sequencing (NGS) have generated large amounts of sequence data at low costs, providing an opportunity to profile STR variations on a population-scale. However, STRs are substantially more difficult to detect based on short reads produced by NGS platform (Gymrek, 2017). Therefore, STR variation analysis has been absent in most whole-genome resequencing studies due to the lack of tools. Recently, numerous methods have been developed to identify STR variants in human, including lobSTR (Gymrek et al., 2012), STRviper (Cao et al., 2014), popSTR (Kristmundsdóttir et al., 2017), and HipSTR (Willems et al., 2017). The program lobSTR was a custom algorithm for genotyping STRs, which has been used to characterize the variation of genome-wide STR loci in Phase 1 of the 1000 Genomes Project and the Simons Genome Diversity Project (Willems et al., 2014; Mallick et al., 2016).

Duck (*Anas platyrhynchos*) is one of the most common domestic fowls that is derived from mallard since 500 BC in central China (Qu et al., 2009). The Pekin ducks as the most elite breed has undergone intensive artificial selection since the Ming Dynasty (A.D. 1368-1644). Compared with their wild ancestors, Pekin ducks show many striking changes such as noble entirely white plumage, an extraordinary big body size and excellent performance of egg production. The Pekin duck is also important model organism for the study of lipid metabolism disorders and immune resistance for viruses, due to its low susceptibility to nonalcoholic fatty liver disease and influenza A viruses infection (Gaidet et al., 2008). Several studies have been performed to develop 100s of duck STR markers (Maak et al., 2000; Huang et al., 2005) and these STRs has also been widely used in researches such as genetic diversity, paternity test, genetic map construction and QTL mapping (Huang et al., 2006, 2007; Su et al., 2007; Ren et al., 2009). However, for the majority of STRs in the duck genome, their population genetics and functional impacts remain poorly defined. Fortunately, the availability of duck reference genomic sequence (Huang et al., 2013) and a dozen of duck genome resequencing data, offer us the opportunity for population scale profiling of duck STRs.

In the present study, we characterized the composition and distribution of STRs in duck reference genome and analyzed the variation of these STRs using genome resequencing in Pekin duck and mallard. Next, we attempt to investigate their population-genetic properties and potential role in duck domestication.

## Materials and Methods

### Samples and Sequencing

A total of 23 mallard and 26 Pekin duck resequencing data were used to profile the STR genotypes. Of which, 21 sequence data were generated in our previous work (Zhou et al., 2018) available at Genome Sequence Archive (GSA)^1^, and the other 28 previously published data were download from National Center for Biotechnology Information (NCBI) Sequence Read Archive (SRA)^2^. We converted the 28 SRA files to fastq format using SRA toolkit (fastq-dump –split-3). All samples were sequenced to an average coverage of 12-fold (range 10–16-fold) with 125PE or 150PE reads on an Illumina Hi-seq (2500 or X Ten) machine. A summary of sample information was presented in Additional File 1.

### Identification of STRs in Duck Reference Genome

The reference genome of Pekin duck used in this study was BGI duck 1.0 reference (GCA_000355885.1), which have been assembled into chromosome level in our previous work based on an RH map (Rao et al., 2012; Zhou et al., 2018). The chromosome level reference genome was available at www.duckbase.org/Download. Tandem Repeats Finder (TRF) was run on the duck chromosomes with a match weight of 2, a mismatch and indel penalty of 7, an 80% probability of matching and a 10% probability of an indel (Benson, 1999). The output was filtered in order to include only repeats with motif length between 2 and 6 base pairs. We removed STRs that localized to areas that might preclude unique mapping, such as large repeats or transposable elements. Transposons and other repetitive elements were identified using RepeatMasker and the TRF results in or within 20 bases of these regions were removed. We furtherly removed STRs with alignment scores below thresholds suggested by Willems et al., STRs located next to or within 20 bases of another STR (Willems et al., 2014). Finally, we successfully assembled the first duck genome-wide STR reference with 198,022 loci included.

### Genotyping STRs in Duck Population

We conducted a comprehensive survey of STR variants using 49 high coverage NGS data from two duck population. LobSTR was applied with default parameters for alignment and STR discovery as previously described (Gymrek et al., 2012). Briefly, we first created a lobSTR reference index based on STR reference using lobstr_index.py script. Then we carried out lobSTR alignment to create the STR alignment bam files, and the final STR variants allelotypes were identified throughout all samples based on the merged alignment file. LobSTR employs an explicit model to enhance accuracy by avoiding stutter noise caused by PCR amplification of a STR locus. The genotyped STR loci were then filtered with the following properties as Gymrek’s recommendation (Mallick et al., 2016): mainly based on coverage, call rate (percent of samples with a genotype call for a given locus), and the metrics Q and DISTENDS reported in the VCF file generated by lobSTR.

• Average coverage < 3 ×
• Average –log10(1–Q) < 0.8
• Call rate < 0.8
• Reference allele length > 80 bp

After filtering loci we additionally filtered individual calls with:

• Coverage < 3 ×
• –log10(1–Q) < 0.8
• Absolute value of DISTENDS score > 20

After filtering, 141,289 loci remained for further analysis.

### Genotyping of SNP in Duck Population

The raw reads were mapped to the reference genome with Burrows-Wheeler alignment (BWA aln) (Li and Durbin, 2009) using the default parameters. Then, the reads mapped to the exact same position on the reference genome were removed with MarkDuplicates in Picard tools to avoid any influence on variant detection. We additionally performed local realignment using GATK (Depristo et al., 2011) to enhance the alignments in regions of insertion-deletion (Indel) polymorphisms. By applying HaplotypeCaller in GATK, we generated a VCF file containing SNPs and short indels. The output was further filtered using VCFtools (version 0.1.15) (Danecek et al., 2011). SNPs that did not pass the following criteria were excluded: (1) the mean sequencing depth (over all included individuals) had to be >3× and <30×; (2) SNPs had to have a minor allele frequency >0.05 and a max allele frequency <0.99; (3) the maximum missing rate was <0.1; and (4) SNPs had only two alleles.

### Primer Design and *in silico* PCR Validation

The 200 bp flanking sequences of each unique STR were extracted for primer design. The Primer3 software^3^ were used and parameters were set as follows: primer length of 18–27 nucleotides, melting temperatures of 55–65°C, GC content of 30–70%, and predicted PCR products of 100–300 bp in length. We further used *in silico* PCR analysis to align primer pairs to reference genome and that matched more than one genome location were removed. The software (e-PCR-2.3.12)^4^ was used for *in silico* PCR analysis with the following default parameters: 50 bp margin, 2 bp mismatch, 1 bp gap, and 50–1,000 bp product size.

### Validating lobSTR Accuracy Using Sanger Sequence and Capillary Electrophoresis

PCR amplifications were carried out in 25 μL reaction mixtures, comprising approximately 50 ng of template DNA, 1.5–2 mm MgCl2 (TaKaRa, Japan), 200 μm of each dNTP, 15 pmol of each primer, and 0.3 U of Ampli *Taq* DNA polymerase (TaKaRa, Japan). Amplifications were performed using the following PCR procedure: an initial denaturation step for 5 min at 95°C, followed by 35 cycles of 95°C for 45 s, 30 s at locus-specific annealing temperature (55–65°C) and 50 s at 72°C, and a final elongation for 10 min at 72°C. The PCR products were subjected to Sanger sequencing to confirm the sequence identity. PCR fragments amplified using fluorescence labeled primer were separated by capillary electrophoresis on ABI 377XL instruments (Applied Biosystems), according to the manufacturer’s recommendations. Genotypes were called using GeneMapperV4.1 software (Applied Biosystems). Details of seven STRs selected for PCR amplifications and its primer information can be found in Additional File 2.

### Correlations of STR Variation With Reference Tract Length and Purity

The heterozygosity of each STR was calculated using the formula 1-Σ^*n*^_*i* = 1_P_*i*_^2,^ where P_*i*_ is the frequency of allele i and n is the total number of alleles. The mannwhitneyu and pearsonr function in python package scipy.stats were used for significant difference and Pearson correlation analysis, respectively. The TRF-reported motif length and purity values were used to categorize the STRs. Repeats were binned by reference tract length, and the mean heterozygosity for each bin was determined to generate plots.

### Principal Component Analysis

We carried out principal components analysis (PCA) using EIGENSOFT software (Patterson et al., 2006) with both STRs and SNPs call set. A Tracy–Widom test was used to determine the significance level of the eigenvectors. We used autosome STR loci with heterozygosity greater than 10% that were called in at least 80% of samples. To encode STRs in bi-allelic format, we followed the convention suggested by Patterson et al. (2006) and encoded each STR allele in the frequency range of 5–95% as a separate bi-allelic marker. This gave 199,999 STR “markers” from 43,939 unique STR loci. The figures were then plotted using the first and second principle components with R packages.

### Evaluation of Genetic Diversity With Polymorphic STRs

The 10% most heterozygous (13,256 loci) autosomal STR loci were used for genetic diversity analysis. To determine whether the two populations had systematically different heterozygosity at these loci, a paired comparison for each STR locus was performed and the cdf function in the scipy.stats.binom python package was used to calculate the *P*-value.

### Functional Annotation of Divergent STRs Using PANTHER and KEGG Database

We formally characterized the function genes containing divergent STRs by searching for overrepresented pathways associated with these genes. Firstly, we extracted the protein sequences for the genes containing divergent STRs. Then, we enriched this genes using “Fasta Protein Sequence” by “Gene-list Enrichment” in KOBAS 3.0 (Wu et al., 2006; Xie et al., 2011). And “Gallus gallus (chicken),” “hypergeometric test/Fisher’s exact test” and “Benjamini and Hochberg (1995)” were chose as “Species,” “Statistical method,” and “FDR correction method,” respectively. Finally, we obtained 9 significant pathways (Corrected *P*-value < 0.05).

## Results

### STR Frequency and Distribution in the Duck Genome

**FIGURE 1:**
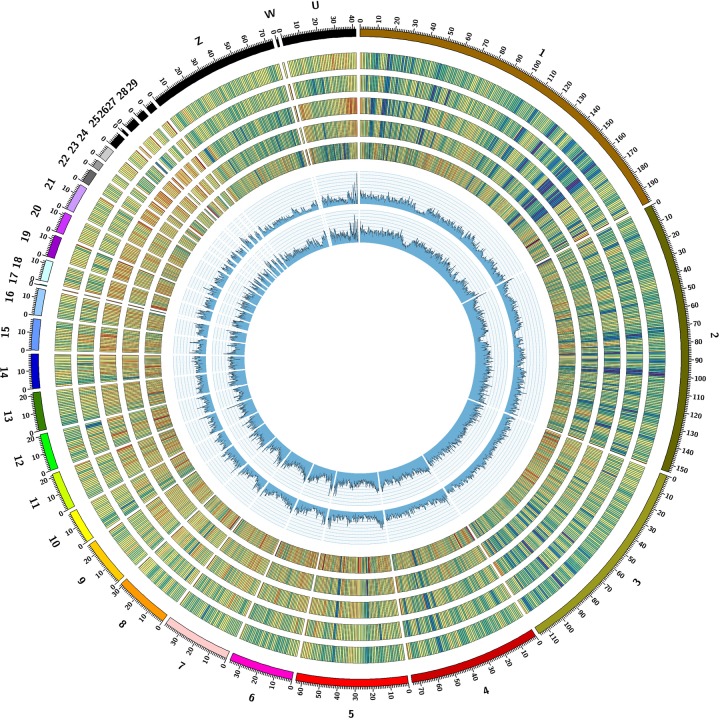
Genomic landscape of STRs in duck genome. Tracks from outside to inside are: I, chromosomes in different colors; II-VI, STR density for Di-, Tri-, Tetra-, Penta- and hexa-nucleotide repeats. STR density in a nonoverlapping window size of 1 Mb is color coded from red to blue, with deeper blue region representing higher STR density. VII and VIII, Heterozygosity of STRs in Pekin duck and mallard was drawn in a 500 kb sliding windows with a 50 kb step, respectively, and STR heterozygosity was averaged for each window.

By screening the genome assembly of Pekin duck, we obtained the first duck STR reference including 198,022 STR loci with repeat motif of di-, tri-, tetra-, penta-, and hexa-nucleotide. The total length of all STR sequences covered 2.10% of the draft genome assembly with an average density of 182.0 STRs/Mb (Table 1 and Figure 1). Among different nucleotide types, the STR frequency was negatively correlated with the number of nucleotide, except tetra-nucleotides. Tetra-nucleotides were the most abundant category, accounting for 38.43% of total STRs, followed by di-nucleotides (27.44%), and tri-nucleotides (16.01%). In contrast, penta-nucleotides and hexa-nucleotides were less frequent compared with others (Table 1). We furtherly examined the nucleotide composition of each motif type and found that some combinations of nucleotides were more prevalent than others in each class. Our analysis showed that STRs with an [A]nT and [A]nC motif were more frequent in each category. Conversely, GC-rich STR motifs were rare in the duck genome. For example, the AT motif was dramatically overrepresented in di-nucleotide motifs, and it was also the most frequent motif in the entire duck genome, which accounting for 12.30% of the total SSR loci discovered. Similarly, the AAT, AAAC, AAAAC, and AAAAAT were the most abundant repeats types in each class. The top three most abundant STR motifs for each class were listed in Table 1, and these STRs represent 82.03, 59.38, 57.64, 40.36, and 21.64% in di-, tri-, tetra-, penta-, and hexa-nucleotide repeats, respectively.

**Table 1:** Distribution and composition of duck STR reference with respect to motif length.

Motif length (bp)	Number of loci	% in reference	Abundance (No./Mb)	Common motifs (% in each category)
Di-	54,347	27.44	49.95	AT(44.81), AC(23.13), CA(14.09)
Tri-	31,711	16.01	29.15	AAT(26.95), AAC(23.40), ATT(9.03)
Tetra-	76,100	38.43	69.95	AAAC(26.91), AAAT(22.52), ATTT(8.21)
Penta-	25,750	13.00	23.67	AAAAC(23.62), AAAAT(10.76), ACAAA(5.98)
Hexa-	10,114	5.11	9.30	AAAAAT(10.61), AAAAAC(7.39), AAAACA(3.64)
Total	198,022	100.00	182.02	

**FIGURE 2:**
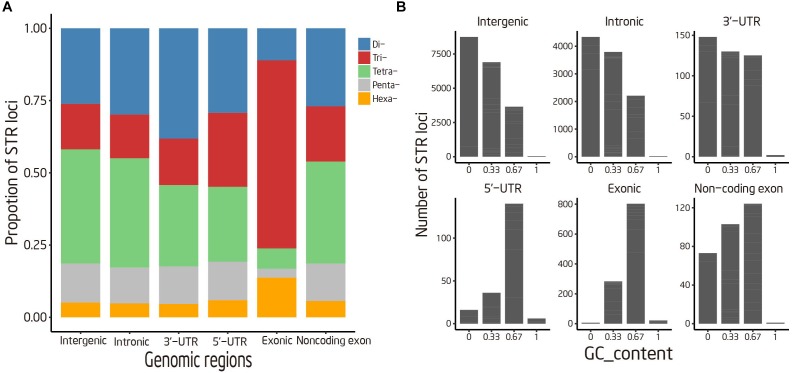
Distribution of STR loci in different genomic regions. **(A)** Percentage of STRs with various motif sizes **(B)** Distribution of tri-nucleotide repeats with various GC content.

STRs were classified into six categories according to its genomic location and annotation of the duck reference genome, to unveil how it was organized in the duck genome. According to our analysis, the majority of STRs were commonly mapped onto intergenic (122,987 STRs) and intronic (68,457 STRs) regions, which together comprised 96.70% of our STR reference (Table 2). There is no apparent difference in STR contents between intergenic regions and introns. Despite low abundance of STRs located in coding region, we found an overrepresentation of Tri- and Hexa-nucleotide repeats in this region (Figure 2A). Moreover, the enriched trinucleotide repeats in exonic and 5′-UTR region were mostly rich in G/C, such as TCC, GAG, and GCA. In contrast to coding regions, A/T-rich trinucleotide repeats were enriched in other genomic regions. However, the trinucleotide repeats containing no “A/T” were quite rare in all genomic regions (Figure 2B).

**Table 2:** Distribution of duck STR in different genomic regions.

Motif length (bp)	Intergenic	Intronic	3′-UTR	5′-UTR	Exonic	Noncoding exon
Di-	32,136	20,409	962	226	189	425
Tri-	19,339	10,355	405	198	1113	301
Tetra	48,620	25,894	709	200	121	556
Penta	16,560	8,503	328	103	52	204
Hexa-	6,332	3,296	116	46	235	89
Total	122,987	68,457	2,520	773	1,710	1,575

### Systematic Profiling of STRs in Duck Populations and Experiment Validation of STR Genotypes

To identify polymorphic STRs in duck populations, whole genome sequence data from 23 mallard and 26 Pekin duck were mapped against reference genome and genotyped using lobSTR. The genomic coverage of sequence data for each animal was around 10∼16× (Additional File 1). In total, 187,874 STRs and 9,354,319 calls were detected in our dataset, with an average STR coverage of 4.8∼7.9×for each sample (Table 3 and Additional File 1). We were not able to genotype about 5% of loci in our reference. Most of these loci have allele lengths greater than 80 bp that could not be spanned by Illumina reads or not suitable for the “allelotype” requirement. Furtherly, we subjected the resulting genotypes to stringent filtering to ensure high quality calls. After filtering, we obtained 141,289 high quality loci with an average length of 19.8 bp, ranging from 11 to 80 bp. Among these STRs, 25,624, 14,944, 36,302, 10,271, and 2,750 STRs were polymorphic (with at least two allele) with repeat motif of di-, tri-, tetra-, penta-, and hexa-nucleotide, respectively (Table 3).

**Table 3:** Overview of STRs genotyped in duck population.

Motif length (bp)	Number genotyped	Number passed filter	Number polymorphic
Di-	52,164	38,519	25,624
Tri-	30,394	22,948	14,944
Tetra-	73,503	58,206	36,302
Penta-	22,881	16,333	10,271
Hexa-	8,932	5,283	2,750
Total	187,874	141,289	89,891

To evaluate lobSTR prediction, we randomly selected 7 loci with motif size of three or four and confirmed the sequence identity of PCR products using Sanger sequencing (Additional File 3). The genotypes of this 7 loci were furtherly validated using Capillary electrophoresis, the gold standard for STR genotyping. We observed that the homozygous STRs with a read coverage of ≥ 3× could be correctly genotyped by lobSTR. For heterozygous STRs, lobSTR may correctly call one allele and miss the other one due to insufficient reads coverage. We observed for homozygous loci 88.89% (40/45) were correctly called whereas the heterozygous showed a lower correct rates of 62.37% (58/93) (Additional File 4).

The full catalog of STR variations is publicly available at http://www.duckbase.org/Diversity in VCF format. In addition, to facilitate the access and effective utilization of the duck STR markers, an integrative table was also available at http://www.duckbase.org/Diversity. Details of each STR, such as location information, repeat type, reference copy number, reference tract length, heterozygosity and locus specific primer pairs could be obtained in the integrative table.

### Patterns of STR Variation

**FIGURE 3:**
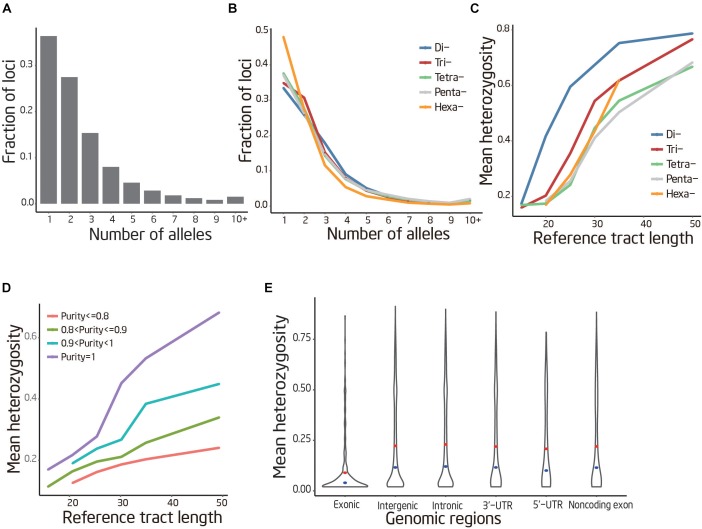
Patterns of STR variation. **(A)** Frequency distribution of the common allele number per locus. **(B)** Frequency distribution of the common allele number per locus stratifying by motif length. **(C,D)** STR variability positively correlated with sequence purity and reference tract length, negatively correlated with motif length. The curves were smoothed by averaging the data points by a sliding window of ± 5 bp. **(E)** Heterozygosity distribution of STR located in different genomic region. The red interior point indicates the median heterozygosity and the blue interior point indicates the average heterozygosity.

The overall trends of STR polymorphism were examined using genotypes identified by lobSTR. Of the 141,289 genotyped loci, 36.1% were observed having more than two common alleles, and some loci have more than 20 common alleles. The remaining loci are either fixed across all individuals (36.4%) or have only two common alleles (27.5%) (Figure 3A). These patterns changed significantly when stratifying by motif length, with longer motif lengths showing less variability. For instance, only 33.5% of dinucleotides are fixed compared to 47.9% of Hexa-nucleotide (Figure 3B).

To examine the relationships between repeat features and variability, heterozygosity of each STR were used as a metric of variation. The present study revealed that heterozygosity depends strongly on properties and genomic locations of the STRs. Our analysis showed that heterozygosity was positively correlated with STR sequence purity (*r* = 0.13, *P* < 10^−100^) and reference track length (*r* = 0.22, *P* < 10^−100^) (Figures 3C,D). In addition, the coding STRs demonstrated significantly decreased heterozygosity compared to noncoding STRs (Mann–Whitney *U*-test; *P* < 10^−10^) (Figure 3E).

### Population Genetic Analyses Using STR Genotype

**FIGURE 4:**
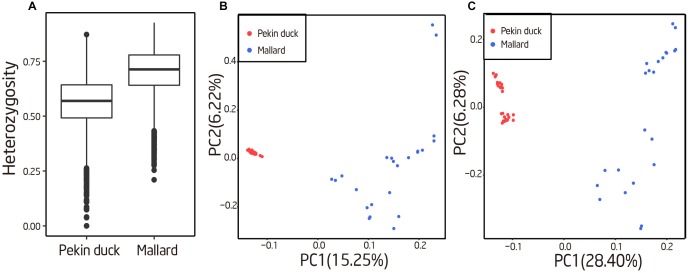
Evaluation of genetic diversity with polymorphic STRs. **(A)** Genetic diversity of the 10% most heterozygous autosomal loci in Pekin ducks and mallards. The box extends from the lower to upper quartiles of the heterozygosity distribution, and the interior line indicates the median. **(B)** The first two principal components based on an analysis of genetic variation at 43,939 autosomal STR loci. **(C)** The first two principal components based on autosomal SNP.

Population genetic properties of mallard and Pekin ducks were explored using the obtained STR genotypes. We first compared the heterozygosities of the 10% most variable autosomal loci between the two breeds, and we found that mallard have a significant higher heterozygosities than Pekin duck (sign test; *P* < 10^−50^), suggesting a higher genetic diversity in mallard (Figures 1, 4A). We performed PCA using the autosome STR loci which have heterozygosity greater than 10%. For comparison, we also performed PCA on autosome SNPs of the same individuals. Mallard and Pekin ducks were clearly separated into two groups on the first principle components from both STR and SNP based PCA Tracy–Widom test; *P* < 10^−26^). Meanwhile, the PCA analysis also revealed higher diversity in mallard than Pekin ducks, which was concordant with the heterozygosities analysis (Figures 4B,C). Our results agree with the known genetic diversity and population structure of the two breeds.

### Population-Differentiated STRs Between Pekin Duck and Mallard

In this study, we identified 89,891 polymorphic STRs in duck population. To investigate the selection signature involved in these STRs, we checked if any STRs were divergent between mallard and Pekin duck by searching for STRs with high degrees of fixation index (*F_ST_*) (Additional File 5). In total, we identified 3122 divergent STRs with a significance level of *P* < 0.01 (*Z*-test). Around 38% of these divergent STR overlap genic elements, i.e., exons, introns, untranslated regions (3′-UTR and 5′-UTR) and noncoding exon, and remaining 62% are intergenic variants. A total of 740 unique genes were found to containing divergent STRs, and an enrichment test was performed to search for significantly overrepresented KEGG pathways (*P_FDR_* < 0.05) using these genes. Nine significantly enriched pathways were identified in this analysis including nervous system and energy metabolism related pathways, such as neuroactive ligand–receptor interaction and ABC transporters (Additional File 6). The conspicuous cluster related to nervous system and energy metabolism implies that these pathways may be involved in the behavioral alternations of duck domestication.

## Discussion

In the last few years, population-scale sequencing projects have made tremendous progress in documenting genetic variation across various vertebrate species. Population scale characterization of STR variation has been reported for humans (Willems et al., 2014), cattle (Xu et al., 2017), and porcine (Liu et al., 2017). Here, we scanned all possible STR loci in duck reference genome and conducted a population-scale analysis of STR variation, providing new insights for further exploration of duck genome.

Our analysis significantly augments the level of knowledge of STR variation in duck population. Before our current study, NCBI has reported only several 100s of STR containing sequence in duck. Our catalog provides the first duck STR reference including 198,022 STR loci with motifs of 2–6 bp. The number of STRs and their density identified in our study was lower than that in human (Willems et al., 2014), which confirmed the prediction that STR abundance is lower in avian than in mammals (Primmer, 1997; Brandstrom and Ellegren, 2008). Without taking of mononucleotide repeats into account, it is generally reported that the most abundant STRs may vary in different organisms, e.g., trinucleotide and dinucleotides repeats were the most common STRs for insect and mammals, respectively (Li et al., 2002). However, the tetra-nucleotide repeats appeared significantly overrepresented in our catalog, which was in agreement with previous reports in other birds (Huang et al., 2016). In addition, our analysis also confirmed the common view that genomic STRs with GC-rich repeats are rare in vertebrate species, such as human, giant panda, and birds (Willems et al., 2014; Huang et al., 2015; Huang et al., 2016).

The distributions of STRs also vary in different regions of a genome. It is well known that noncoding regions generally contain more abundant STR than coding regions. Despite the relative lowest abundance of STR in exons, a propensity of tri- and hexa-nucleotides in exons were observed in our study, which was in agreement with observation in human (Willems et al., 2014) and giant panda (Huang et al., 2015). These STR loci can vary without introducing frameshift mutations and therefore may be exposed to weaker purifying selection. Moreover, the GC-rich trinucleotide repeats were also found to be enriched in exon and 5′-UTR region. This observation suggests that the occurrence of trinucleotide repeats in exon and 5′-UTR region is not random, but undergoes positive or negative selective pressure. Previous studies have reported that GC-rich trinucleotide repeats are capable of forming stable hairpin or quadruplex structures and are involved in the regulation of transcription (Jasinska et al., 2003; Kozlowski et al., 2010).

Based on the defined STR reference catalog, our population-scale analysis of STR variation obtained genotypes for 187,874 STR loci. After filtering, 89,891 polymorphic STRs were obtained, which could be useful genetic markers for future studies such as parentage analysis, genetic diversity and QTL mapping. It has been reported that STR variability were determined by its motif characteristics in human (Willems et al., 2014) and *Drosophila melanogaster* (Fondon et al., 2012). Our study confirmed the well-known relationship that shorter repeat motif, longer reference tract length, higher purity, and residing outside of a coding region are all associated with an increase in STR variability (Pemberton et al., 2009; Fondon et al., 2012; Mallick et al., 2016). The overall positive relationship between repeat feature and polymorphism observed here suggests that STR loci with shorter repeat motif and longer repeat length mutate more rapidly. Clearly, for any given total length of a STR loci, interruptions have the consequence of lowering variability, most likely due to the stabilizing effects of unique sequence within tandem array that prevent replication slippage (Petes et al., 1997; Rolfsmeier and Lahue, 2000; Sibly et al., 2003).

It is well known that most domesticated animals have experienced a “domestication bottleneck” with reduced genetic diversity relative to their wild ancestor(s) (Chen et al., 2007; Gibbs et al., 2009). STRs are important molecular markers for studying genetic diversity and population structure (Tadano et al., 2014; Abebe et al., 2015; Santos et al., 2016). The genome-wide STR genotypes were used to evaluate genetic diversity of Pekin duck and its ancestor (mallard). A higher genetic diversity was observed in mallard than Pekin duck, which suggest that the current genetic diversity of the wild population could be important genetic resource for future breeding programs. Previous study has reported that genome-wide STR genotypes could be used to distinguish population structure and divergence patterns among population groups (Sonay et al., 2015). We performed principal component analysis (PCA) based on STR genotypes and found that mallard and Pekin ducks could be clearly separated into two groups on the first principle components. These STR markers could then potentially be used for conservation and breeding programs of Pekin ducks, since their higher polymorphism information content means that a low number of markers can be used.

Pekin ducks have experienced natural and artificial selections for 1000s of years, and these selections has resulted in striking changes in traits such as boy shape, color, behavior, and reproduction (Qu et al., 2009). However, the molecular mechanisms underlying the selection-causing phenotypic changes in Pekin duck remained largely unknown. To reveal the potential contributions of STRs in the process of duck domestication, we carried out a comparative study to identify the STRs with extreme divergence in allele frequency (*F_ST_*) between Pekin duck and mallard. A total of 3,122 divergent STRs were obtained, which overlapped with a set of genes related to nervous system, energy metabolism and behavior. Of the significantly enriched pathways, neuroactive ligand–receptor interaction was found to have 30 genes containing divergent STRs. The enriched genes were also including growth hormone receptor (*GHR*) and thyroid stimulating hormone receptor (*TSHR*), which has been well established to play pivotal role in growth and metabolic regulation of chicken. It was also worth noting that *TSHR* was also located in a selected region as our previous reports (Zhou et al., 2018). *GHR* has been reported to contain a deletion in its exon as causative mutation for sex-linked dwarfism. And this mutation has been widely used in breeding of commercial broiler lines to improve the feed conversion rate (Agarwal et al., 1994). *TSHR* has been identified previously as domestication gene for chicken, and related to the absence of the strict regulation of seasonal reproduction found in natural populations (Yoshimura et al., 2003). This results are in accordance with previous reports that strong selection on behavioral alternations and improve reproduction ability are the common characteristics of animal domestication such as chicken (Rubin et al., 2010), dog (Axelsson et al., 2013), and yak (Qiu et al., 2015).

## Conclusion

In this study, we analyzed the composition and distribution of STRs in the duck reference genome and investigated the population-genetic properties of these STRs using genome resequencing on 23 mallard and 26 Pekin ducks. We confirmed the well-known relationship that STR polymorphism was closely related to its genomic location, motif length, purity and repeat number. The population genetic analysis suggests that STR genotypes obtained from genome resequencing data could be used to distinguish population structure and divergence patterns among population groups. Our results also revealed that the genes overlapping with divergent STRs perhaps involved in domestication of duck by influencing the nervous system and energy metabolism. Altogether, our study provides valuable resource for future study of genetic diversity and genome evolution in duck.

## Ethics Statement

All experimental procedures with ducks were performed according to the Guidelines for Experimental Animals established by the Ministry of Science and Technology (Beijing, China). Ethical approval on animal survival was given by the animal ethics committee of the Institute of Animal Sciences (IAS), Chinese Academy of Agricultural Sciences (CAAS, Beijing, China) with the following reference number: IASCAAS-AE-03.

## Data Availability

The Illumina sequencing data used in this study can be available at (SRA, https://www.ncbi.nlm.nih.gov/sra) and (GSA, http://gsa.big.ac.cn). The accession numbers for each sample are included in the Additional Files. Other data sets supporting the results of this article are included within the article and its Additional Files.

## Author Contributions

ZZ and SH conceived and coordinated the study. WF performed the study and wrote the manuscript. WF, LX, HC, and ML carried out the bioinformatics and experiments analyses. LX, HL, YJ, and YG gave advice about concept and revised manuscript. All authors read and approved the final manuscript.

## Conflict of Interest Statement

The authors declare that the research was conducted in the absence of any commercial or financial relationships that could be construed as a potential conflict of interest.

## Footnotes

1 http://gsa.big.ac.cn

2 https://www.ncbi.nlm.nih.gov/sra

3 http://bioinfo.ut.ee/primer3/

4 ftp://ftp.ncbi.nlm.nih.gov/pub/schuler/e-PCR/

## Supplementary Material

Additional File 1 Summary of sample information used in this study.

Additional File 2 Details of seven STRs selected for Capillary electrophoresis and its primer informations.

Additional File 3 Sanger sequence of PCR products from seven selected STR.

Additional File 4 Comparison of capillary electrophoresis and lobSTR results for seven Selected STRs.

Additional File 5 Chromosome-wise deviation in STR constitutions based on FST.

Additional File 6 Significant KEGG pathways enriched by genes containing divergent STR.
