# Supplementary material for: Characterization of Duck (Anas platyrhynchos) Short Tandem Repeat Variation by Population-Scale Genome Resequencing
Source: Front Genet. 2018 Oct 30;9:520. doi: 10.3389/fgene.2018.00520 (PMC6218588; doi:10.3389/fgene.2018.00520)
Supplement: Additional File 3 — Sanger sequence of PCR products from seven selected STR. [file Table_3.DOCX]

>IAS01_refseq

ACTAGGGAACAAGGCAGGGTTTCCCAGGATTGCCTTTCAAATATGCACTCCAACTCTATACCTGTGCGGGCACATGGCGTTTTTGATGTTGCAAGTTCTTTGACATGGGGAAAGGTGTCGTTAATGAGTTCTTCATTATTATTATTATTATTTATTTCCCCAGAAAAGTAGATTTTACTTTATGGGCTCAGATATGATCCCTACTCTTCCCTTCATACCAGAGAGAGCTGGTAACTG

>IAS01_CRX022265

CGGTAGCATCATATGCACTCCACTCTATACCTGTGCGGGCACATGGCGTTTTTGATGTTGCAAGTTCTTTGACATGGGGAAAGGTGTCGTTAATGAGTTCTTCATTATTATTATTATTATTATTATTATTATTATTATTATTATTTATTTCCCCAGAAAAGTAGATTTTACTTTATGGGCTCAGATATGATCCCTACTCTTCCCTTCATACCAGAGAGAGCTGGTAACTGGGTGTTAGCCACCTAACTTGGGCTCAGTAGAAGACCGAGTAAGCCTCTGTATGTTGCATTGGTCTGGGTGGGAGAGA

>IAS01_CRX022262

CCGGTATGCATTCATATGCACTCCACTCTATACCTGTGCGGGCACATGGCGTTTTTGATGTTGCAAGTTCTTTGACATGGGGAAAGGTGTCGTTAATGAGTTCTTCATTATTATTATTATTATTATTATTATTATTATTATTTATTTCCCCAGAAAAGTAGATTTTACTTTATGGGCTCAGATATGATCCCTACTCTTCCCTTCATACCAGAGAGAGCTGGTAACTGGGTGTTAGCCACCTAACTTGGGCTCAGTAGAAGACCGAGTAAGCCTCTGTATGTTGCATTGGTCTGTGTGGGAGAGAT

>IAS03_refseq

AAACACTTTGGGGCTTTTTGGCAGTGTGTATTATTTAGGTGGCAAAAGCACTGTGCCCCGATGTTAACAACTAGTTAAATCACAGAAGAGTACAGGTTCCTAGAAAACACTTGACTCTTAATTTTCACCCACTTATAAGCTTGTCACTAAGTCTCATACTGAAGGTACTGTCATAAGGACAATTTATTATTATTATTATTATTATTATTTTAACCTTCAAGTGTATAATAAGCCAAA

>IAS03_CRX022272

TTTGGATTACGTGGCAAGCACTGTGCCCCGATGTTAACAACTAGTTAAATCACAGAAGAGTACAGGTTCCTAGAAAACACTTGACTCTTAATTTTCACCCACTTATAAGCTTGTCACTAAGTCTCATACTGAAGGTACTGTCATAAGGACAATTTATTATTATTATTATTATTATTTTAACCTTCAAGTGTATAATAAGCCAAAGCTAACATGCACTGCTCTTAATTAAAGATAATCTATAACAAAACATAAGACCACTTTAAAGCAATTTGTGTTGCTTCTAGTTGTATGCAGCTATAAGAATTTACTGCATTACCCTGTGCTACTTTCTGATGTGATGAAAATCGGTGTGG

>IAS03_CRX022273

AGGGTATTTTCGTGGCAAAGCACTGTGCCCCGATGTTAACAACTAGTTAAATCACAGAAGAGTACAGGTTCCTAGAAAACACTTGACTCTCAATTTTCACCCACTTATAAGCTTGTCACTAAGTCTCATACTGAAGGTACTGTCATAAGGACAATTTATTATTATTATTATTATTATTATTATTATTATTATTATTTTAACCTTCAAGTGTATAATAAGCCAAAGCTAACATGCACTGCTCTTAATTAAAGATAATCTATAACAAAACATAAGACCACTTTAAAGCAATTTGTGTTGCTTCTAGTTGTATGCAGCTATAAGAATTTACTGCATCTGTGCTACTTTCTGATGTGATGAAATCGGTGTGG

>IAS06_refseq

AGGCAGAGGTCAACTCCTCACTACCAAAGAATCCCAGGCTGAATCCTCATATACTAAGGACAAAACACACTGCGTGAGATCCCCTCTTAGGCCAGGCCAGCTGTGTTTCCTGTAGCCAAGCACTGCAGTAATGAAGGGAGTTTTTCCTATGGACAAAGCTCTTGGACTACTCGATAGAGGAATTTAAAAATCCTTTTGTTGTTGTTGTTGTTGTTTAGGGTGTTGCTTTCAGTGAGG

>IAS06_CRX022276

TCCAAGCTGATCCTCATATACTAAGGACAAAACACACTGCGTGAGATCCCCTCTTAGGCCAGGCCAGCTGTGTTTCCTGTAGCTAAGCACTGCAGTAATGAAGGGAGTTTTTCCTATGGACAAAGCTCTTGGACTACTCGATAGAGGAATTTAAAAATCCTTTTGTTGTTGTTGTTGTTGTTGTTGTTGTTGTTTAGGGTGTTGCTTTCAGTGAGGTGG

>IAS06_CRX022274

CCAGGCTGATCCTCATCTACTAAGGACAAAACACACTGCGTGAGATCCCCTCTTAGGCCAGGCCAGCTGTGTTTCCTGTAGCCAAGCACTGCAGTAATGAAGGGAGTTTTTCCTATGGACAAAGCTCTTGGACTACTCGATAGAGGAATTTAAAAATCCTTTTGTTGTTGTTGTTGTTGTTGTTGTTGTTGTTGTTGTTGTTGTTGTTGTTGTTGTTTAGGGTGTTGCTTTCAGTGAGGTGG

>IAS07_refseq

AATTGGCTGTTCCCAAAGTGCAGGCTGTTTCTTACTACAAGTGTAGCTGAATCACTTGTTTACTCACATCTCTCTTTGTTTTATTTTTAAGACTTTTGAATGAACTTTAAAAAATGATTTTGGGAAATCTGTTTGAGGTAAAATTTCATATCAGGACTCCTTTGTCAGTGGTGATAGATAGATAGATAGATAGATGGTACAAAGATGTTGCAGTGAGGAAGC

>IAS07_CRX022281

CAGCTGTAGCTGATCACTTGTTTACTCACATCTCTCTTTGTTTTATTTTTAAGACTTTTGAATGAACTTTAAAAAATGATTTTGGGAAATCTGTTTGAGGTAAAATTTCATATCAGGACTCCTTTGTCAGTGGTGATAGATAGATAGATAGATAGATAGATAGATAGATAGATAGATGGTACAAAGATGTTGCAGTGAGGAAGCAA

>IAS07_CRX022267

CAAGGGTAGTGTAGCTGATCCTTGTTTACTCACATCTCTCTTTGTTTATTTTTAAGACTTTTGAATGAACTTTAAAAAATGATTTTGGGAAATCTGTTTGAGGTAAAATTTCATATCAGGACTCCTTTGTCAGTGGTGATAGATAGATAGATAGATAGATAGATAGATAGATAGATGGTACAAAGATGTTGCAGTGAGGAAGCAA

>IAS12_refseq

TTGAGGGCATTGACTTGTTGTAAATAATTTCTAAAGATTAACTATTATTTGAGATGATTTGTCATCCTTCATGCTGAGACTCTCTGTCAAGAAAGAAGGAAATAAGTACTTCAGTCAAGCCATTTGTTGTAGTTGCAAGCAAATGTAATAGTTTTTATTTATTTATTTATTTATTTATTTATGGGAGGACAGTTGGAGATTTGCTTAAGTGAAGTGTCTGGAGACAAAGATCCTGGA

>IAS12_CRX022277

CTTAGATACTATTATTTGAGATGATTTGTCATCCTTCATGCTGAGACTCTCTGTCAAGAAAGAAGGAAATAAGTACTTCAGTCAAGCCATTTGTTGTAGTTGCAAGCAAATGTAATAGTTTTTATTTATTTATTTATTTATTTATTTATTTATTTATGGGAGGACAGTTGGAGATTTGCTTAAGTGAAGTGTCTGGAGACAAAGATCCTGGAAGTTAAACTCCTTTTCCCTAGAGAGTGTTTTCTCATAAACTGTAGAGCACAATAACTGTCTTCTGGTCTAGCAAATGATAGCTGTCATTCGTATCACAAGCTTAGTTTGTTTTGAAATGTGCTTTGTATTCTGTTGGA

>IAS12_CRX022278

AGATCAGATACTATTATTTGAGATGATTTGTCATCCTTCATGCTGAGACTCTCTGTCAAGAAAGAAGGAAATAAGTACTTCAGTCAAGCCATTTGTTGTAGTTGCAAGCAAATGTAATAGTTTTTATTTATTTATTTATTTATTTATTTATTTATTTATTTATTTATTTATTTATGGGAGGACAGTTGGAGATTTGCTTAAGTGAAGTGTCTGGAGACAAAGATCCTGGAAGTTAAACTCCTTTTCCCTAGAGAGTGTTTTCTCATAAACTGTAGAGCACAATAACTGTCTTCTGGTCTAGCAAATGATAGCTGTCATTCGTATCACAAGCTTAGTTTGTTTTGAAATGTGCTTTGTATTCTGTTGGA

>IAS13_refseq

TCCCTGATCTAAGGTCCCTGGAAATACCTGAAAAAAGTCTGTGACCATTAGTCCACAAAATGAAAATTGCTTTCTCTTATGTCATTACGTAGGAACGAACTCATCCCACTATTTTAATGTATCTGTGTATCTTCAGCCTCAGTGGATTTTTGCAAGAATGCCACAATTTTCTGAACTGTCATATGTTTTATTATTATTATTATTATTATTATTATGTTTTTTAATATGCTTTCAGTG

>IAS13_CRX022262

AAAAGTCTGTGACATTAGTCCACAAAATGAAAATTGCTTTCTCTTATGTCATTACGTAGGAACGAACTCATCCCACTATTTTAATGTATCTGTGTATCTTCAGCCTCAGTGGATTTTTGCAAGAATGCCACAATTTTCTGAACTGTCATATGTTTTATTATTATTATTATTATTATTATTATTATTATTATTATTATGTTCTTTAATATGCTTTCAGTGATTCTTTGTTGCCTTGAA

>IAS13_CRX022279

GGCAAGTCTGTGACATTAGTCCACAAAATGAAAATTGCTTTCTCTTATGTCATTACGTAGGAACGAACTCATCCCACTATTTTAATGTATCTGTGTATCTTCAGCCTCAGTGGATTTTTGCAAGAATGCCACAATTTTCTGAACTGTCATATGTTTTATTATTATTATTATTATTATTATTATTATTATTATTATGTTCTTTAATATGCTTTCAGTGATTCTTTGTTGCCTTGAA

>IAS14_refseq

CAACTACTCCGTGGTCAGCAGCTACAGGAAAGAACTTCCTTTCAGAGTAAATTTTGGTTGGAGTATCTCTCGAAAGAGTCAAGAAACTTCTTTGGTACAATTTTGTTACAATTAATAATTTCCAAATTCTAATTCATTTGCCAGATATTGAAAATAAATAAATAAATAAATAAATAAAAAGTGCACATCTTATCAGGTAAATGGAAGAAAAAGATACATGAGATCAGGGAAGGATTT

>IAS14_CRX022275

AGAACTCATTTCGAGTAATTTTGGTTGGAGTATCTCTCGAAAGAGTCAAGAAACTTCTTTGGTACAATTTTGTTACAATTAATAATTTCCAAATTCTAATTCATTTGCCAGATATTGAAAATAAATAAATAAATAAATAAATAAATAAATAAAAAGTGCACATCTTATCAGGTAAATGGAAGAAAAAGATACATGAGATCAGGGAAGGATTTTGTAATGTGCTTCTTGTTCAGTTCTACCAAAATTGTGCTTGATACTGGAGATTTTGGAAAGTCTTCAGTTTATTTGCTTGGTATTTACCTGGTACTTAATATTAATGTTGGGAGTAGAGGTCGTTGCTAA

>IAS14_CRX022276

GGTGACTCTTTCGAGTAATTTTGGTTGGAGTATCTCTCGAAAGAGTCAAGAAACTTCTTTGGTACAATTTTGTTACAATTAATAATTTCCAAATTCTAATTCATTTGCCAGATATTGAAAATAAATAAATAAATAAATAAATAAATAAATAAATAAATAAATAAAAAGTGCACATCTTATCAGGTAAATGGAAGAAAAAGATACATGAGATCAGGGAAGGATTTTGTAATGTGCTTCTTGTTCAGTTCTACCAAAATTGTGCTTGATACTGGAGATTTTGGAAAGTCTTCAGTTTATTTGCTTGGTATTTACCTGGTACTTAATATTAATGTTGGGAGTAGAGGTCGTTGCTCA
